# Do expert assessments converge? An exploratory case study of evaluating and managing a blood supply risk

**DOI:** 10.1186/1471-2458-11-666

**Published:** 2011-08-24

**Authors:** John Eyles, Nancy Heddle, Kathryn Webert, Emmy Arnold, Bronwen McCurdy

**Affiliations:** 1 School of Geography and Earth Sciences, McMaster University, 1280 Main St West, Hamilton, Ontario, Canada; 2 Transfusion Medicine Program, McMaster University, 1200 Main St West, Hamilton, Ontario, Canada

## Abstract

**Background:**

Examining professional assessments of a blood product recall/withdrawal and its implications for risk and public health, the paper introduces ideas about perceptions of minimal risk and its management. It also describes the context of publicly funded blood transfusion in Canada and the withdrawal event that is the basis of this study.

**Methods:**

Interviews with 45 experts from administration, medicine, blood supply, laboratory services and risk assessment took place using a multi-level sampling framework in the aftermath of the recall. These experts either directly dealt with the withdrawal or were involved in the management of the blood supply at the national level. Data from these interviews were coded in NVivo for analysis and interpretation. Analytically, data were interpreted to derive typifications to relate interview responses to risk management heuristics.

**Results:**

While all those interviewed agreed on the importance of patient safety, differences in the ways in which the risk was contextualized and explicated were discerned. Risk was seen in terms of patient safety, liability or precaution. These different risk logics are illustrated by selected quotations.

**Conclusions:**

Expert assessments did not fully converge and it is possible that these different risk logics and discourses may affect the risk management process more generally, although not necessarily in a negative way. Patient safety is not to be compromised but management of blood risk in publicly funded systems may vary. We suggest ways of managing blood risk using formal and safety case approaches.

## Background

### Introduction

Blood is a special product, being integral to life and inside our bodies. As Chan [1] notes (following Titmuss [2]), there is much symbolism associated with blood. As a medical solution to a health problem it depends on the gift of others. Its gift or donation is altruistic and seen as sharing what is essential to life. In Christian societies, blood is seen as having the ability to wash away sins [3]. It represents purity, intensified by its gift to others. Blood donation is a very visible connection to others, even in societies where payments are made to donors [4]. These payments remain controversial and may lead to compromises in the safety of blood products. Blood donation is indeed a two-edged sword: a lifesaver or a transmitter of disease. The struggle over successful compatibility of blood types and to combat transmissible diseases has been a long but largely successful story [5]. The hazards in receiving transfused blood were heightened by HIV/AIDS, which as Chan [1] argues, led to the blood risk being intensified by this stigmatising illness. But since the mid-1980s, screening tests for sexually transmitted infections, including HIV and, later, other transmissible diseases have been implemented in many countries worldwide. In fact, scientific risk assessments, the vigilance of existing or new federal agencies, enhanced public information and disclosure have greatly improved both the calculated and perceived risk of 'bad blood'. As we shall see, Canada is no exception but it is a jurisdiction where there is particular salience.

Blood transfusions are overwhelmingly safe, with about 0.5 to 3 percent of all transfusions resulting in adverse consequences [6]. A proportion of these adverse events result from error in preparation and administration. Other adverse events are classified as infectious (e.g. HIV/AIDS, hepatitis, human T-Cell lymphotropic virus (HTLV), West Nile virus) or non-infectious (these are commoner and include acute haemolytic reaction, transfusion associated acute lung injury, allergic reactions, graft-host disease). While severe non-infectious complications are rare, case fatality is high. Infectious disease risks associated with blood transfusion in Canada are currently estimated at 1 per 7.8 million units transfused for HIV, 1 per 2.3 million for hepatitis C, and 1 per 153,000 for hepatitis B [7]. For manufactured plasma-derived products, the risk is estimated at less than 1 in 10 million or theoretical [8]. Bacterial infection from contaminated blood products is also possible but routine bacterial screening of high risk products (platelets) and changes in blood collection techniques have reduced this risk significantly. Methods to inactivate pathogens in blood products (those currently known and future threats), have been developed for plasma and platelets and methods applicable to red cells are under development. Canadian blood suppliers are currently exploring the feasibility of implementing these new technologies once they are available [9]. So despite public concern (see Lee [10]), experts would surely see risk as minimal and theoretical. And they do. But this does not prevent every 'unsafe' blood incident from being rigorously pursued. But this assumes that all experts may have the same goals or different pathways to similar goals which may shape the treatment of the hazard differently. In part they do - the safety of the product and public health. But do different groups of experts have other goals or interpretations which may shape the treatment of hazard in specific ways? This is the broad question explored in this paper, the major contribution of which is to examine the divergence of expert opinion even where science is consistent and the goal universal.

### A Constructivist View of (Blood) Risk

To answer our question, a constructivist approach is adopted. Assessing and managing all kinds of risk is a challenging task but this is especially the case in those risks pertaining to human health which are largely dealt with in the public domain. Because these concerns do invoke public discourse, much attention has been paid to the differences, if any, between expert and public perceptions and assessments [11]. For twenty years or more, constructivist analyses have brought to the fore the importance of prior social, cultural, institutional and political factors that shape and are embedded in both lay and expert risk assessments (see [12]). Lay people and experts use similar cognitive processes with both falling prey to errors from anchoring, overconfidence and the gambler's fallacy (see [13]) Attention has also turned to the perceptions and assessments of experts themselves. Van Zwanenberg and Millstone [12] comment that the construction of scientific claims can inform risk assessments. Furthermore, a "coherent realm of expertise" may not exist, as Haggerty [14:201] points out with respect to crime risk. He adds that expert opinion about crime risk is incredibly fractured with there being differences in professional opinion with respect to dimensions of high-profile risks as well as the nature and efficiency of crime prevention. Sjoberg [15] also points out that there is likely to be a whole range of expertise and this range is not well-articulated in many risk perception studies. If, therefore, there is this range of expertise, why should we expect expert opinions to converge, although overconfidence in technical and medical assessments may exist (but see [16])? In fact, we shall note certain biases in expert opinion.

Shanteau [17] questions the convergence of expert opinion which is based on the widespread use of statistics and economics in assessments and on the widespread search for generalizability in how people think about risks. Shanteau argues then that the bases of convergence are in themselves flawed (see also [18-20]). Of course, it is recognised that different risk logics or discourses - i.e. different conceptions of an activity or event as risk - exist, but these have seldom been applied to expert opinion (see [21,22]). Silva et al. [23] show, in an experimental setting, that despite differences in scientific background and political beliefs, scientists tend towards a precautionary stance over the setting of safety standards. Yet scientists are not the only type of expert involved in such matters. McMahan et al., [24] point to the differences in how scientists and risk assessors regard electric and magnetic fields. Furthermore, Chalmers et al. [25] show how the opinions of dentists and nursing directors vary over dental care in nursing homes. Chociolko [26] provides an example of expert disagreement often found in sometimes adversarial settings in which experts 'take sides' representing, say, industry or community.

How might we assess expert opinion and convergence on risky matters? Shanteau [17] points to the importance of the level of decision, made by experts using a medical analogy of diagnosis (what is it?), prognosis (what is the likely outcome?) and treatment (what to do about it?), with a different logic applied on different levels. Furthermore, experts and professionals possess similar cognitive properties as non-experts. "Experts are not immune to the cognitive illusions that affect other people" [27]. And it is increasingly noted that cognition and emotion are intertwined in decision-making (see [28]). As Cross [29:28] comments: "when an attitude or an activity is of considerable importance to a person, the individual is loathe to believe that it is hazardous." Furthermore, biases or heuristics found in studies of perceived risk may be found among experts and affect their assessments and opinions. In this paper, we address some of these issues about expert opinions, utilising the ideas of non-convergence (especially at different levels of an assessment), and of the heuristics made by different professional groups (commitment to a particular practice, reliance on rational models and thinking), to understand different concepts of risk relating to the blood supply arising from a case when supply of 'safe blood' was potentially disrupted in one Canadian city. So all may want 'safe blood' but do the management and decision-making styles and discourses of all actors converge or vary?

### The Context and the Case

'Safe blood' has been a major policy issue in Canada. This is also a sensitive issue, which likely arises from the 'tainted blood tragedy' of the 1970s and 1980s. At that time, the blood supply was organised and managed by the Canadian Red Cross Society (CRCS) as this body had been responsible for blood supply during the Second World War. CRCS is a not-for-profit, humanitarian society, with many diverse activities extending beyond the responsibility for the blood supply which are largely carried out by volunteers. A chronic lack of funding for the CRCS held back technical developments to collect and supply blood in Canada. Government funding only began to increase in the mid-1970s as procedures for blood collection became modernised. But its principles as part of the international Red Cross (non-discrimination against individuals and independence from government) meant its screening and management procedures became fatally flawed in the Canadian context, with often strained relations between volunteer oversight and professional activities. These limitations set the stage for tragedy with the discovery of blood-borne diseases as recruitment of donors was still in volunteer hands and carried out with self-answered questionnaires. The recipe for disaster was set, and the situation was worsened by the failure to track those who had received tainted blood and a failure to apologise or compensate affected individuals on the parts of the provincial and federal governments (see [30,31]). As Picard [30] has noted, 'tainted blood' was arguably the largest public health catastrophe in Canada. "About 1,000 individuals who received blood transfusions in Canada between the late 1970s and 1980s were infected with HIV and another 30,000 were infected with hepatitis C" [32]. Eventually, this led to the establishment of a public inquiry in 1993 with the final report of the Krever Commission being delivered in 1997 [31]. Krever recommended the creation of a new blood system and the Commission's recommendations led to the creation of Canadian Blood Services to operate the system in all provinces, except Quebec which formed its own agency, Héma-Québec. With the creation of these agencies, the Canadian blood system joined the U.S. and West European countries in having an expert-based and scientifically-based system and CRCS went on to different humanitarian tasks.

For the blood system and perhaps other aspects of the Canadian healthcare system, Krever highlighted two key elements - the institution of precautionary measures and the creation of a governance system emphasising safety (see [32]). Risk assessment became based on scientific tests and evidence and the management system highly coupled and structured to protect human health. The Canadian blood system is currently seen as a high reliability organisation, emphasising safety and responsiveness to problems [33]. In fact, these elements were introduced so the Canadian blood system could respond to threats in a timely and effective way. This was the case for threats from infectious agents with product recall or donor deferral being rapidly instituted for variant Creutzfeldt-Jacob disease (See [33,34]), West Nile virus (see [35]) and SARS. It is also the case for 'organisational threats' in which practice errors over labelling, documentation, or other deviations from procedures are dealt with through product withdrawal. For such threats, it may be said that the Krever report is one dimension in the increasing use of quality assurance for among other things patient safety in health care. But we know from other settings that challenges occur and 'accidents' (often resulting from operator error or technical malfunction) are normal in complex systems [36]. It seems therefore worth discovering if in this highly coupled, apparently well-managed system, all experts agree about the nature of arising hazards.

Recalls involving small numbers of blood products occur frequently (daily), for example, when donors provide post-donation information that affects their eligibility as a blood donor. Whereas withdrawals, which occur less frequently, are often due to operational deviations where the risk is minimal or unknown and may involve a large number of blood products. Withdrawal involving a large number of blood products can jeopardize the availability of an adequate blood supply.

When there is a real or perceived threat to the safety of the blood supply, withdrawals occur without reference to cost or potential short-term shortages. A major withdrawal of blood products in Ontario in 2005 involved over 3500 blood product components. The reason for this withdrawal was clerical in nature associated with records of donation which are completed at the time each donor gives blood. The risk to the blood supply was likely minimal or nonexistent. Individuals in the appropriate organizations joined forces to deal with the matter. But it brought to light many of the challenges faced by both hospitals and the blood suppliers around dealing with error and emphasized gaps in the system related to timeliness; communication; risk perception; and recipient notification. It provides an example of what we call organizational 'threat' or, more positively, a learning opportunity. We suggest that this approach to identifying error as a potential for hazard and risk is useful in that it focuses attention on possible differences in organizational responses and public notification.

So given the context, it would appear that the case could have been dealt with procedurally with all parties - regulators, blood product supplier, hospitals and treatment facilities and agents - being on the same page. The risk from the blood product should have been seen in a universal way by all parties involved in this particular case. But was it? If there are different levels of decision-making and risk assumptions do expert assessments necessarily converge? The answer, to anticipate, is yes and no.

## Methods

This paper is an outgrowth of a study undertaken to determine the current procedures and protocols for handling the recall/withdrawal of blood products in Ontario and beyond [37,38]. In this we adopt a quasi-foundational position, with the experiences and views of respondents being seen in the answers to our questions (see Additional File 1: Appendix 1) from which exemplars in the form of quotations are drawn (see [39]). We undertook a number of one-on-one interviews with key stakeholders in the blood supply and management arena, both within and beyond Ontario. Interviews were conducted with individuals from Canadian Blood Services, Héma-Québec, Ministry of Health and Long Term Care (Ontario), relevant staff at hospitals and hospital laboratories, as well as blood product recipients. These recipients were not professional or credentialized experts but it was felt that their experience of the blood transfusion system might add a different perspective to our investigation. Within hospitals, we interviewed physicians (both transfusion medicine physicians and physicians who do not work in the transfusion service but who use blood products), hospital administrators, CEOs, risk managers and public relations personnel. Within hospital laboratories, we interviewed managers, technologists and transfusion safety officers. In other words, we interviewed in total 45 professionals involved in blood supply - regulators, suppliers, treatment individuals and agencies, administrators, laboratory personnel and transfusion recipients. The project's steering committee (composed of individuals from the blood supplier, hospitals and laboratories) helped to identify individuals from the blood supply and management arena who had experience of blood product recalls and withdrawals and who could therefore be anticipated to be able to provide rich data. This purposeful sampling was augmented by asking those individuals interviewed to suggest others whom they thought it would be helpful to interview. We employed a multilevel sampling framework in that we wished to compare the perceptions of those with potentially different stances on blood risk management (see [40]). We recognise that the sample size in our study limits the generalisability of our findings and interpretations, and it may indeed be exploratory with our practice suggestions being essentially that--suggestive. Table 1 shows the number of participants interviewed within each category. As well as interviews, copies of written relevant rules and procedures pertaining to recalls/withdrawals were obtained. And we may ask - does opinion converge on the type and nature of the risk?

**Table 1:** Job categories of respondents in blood risk study

Category #	Job title	# of participants interviewed
1	Laboratory Manager	3

2	Transfusion Safety Officer	2

3	Lab Technologist (Blood Bank)	9

4	Physicians - Blood Bank	4

5	Physicians - who use blood products	7

6	Hospital Administration	4

7	Hospital PR	1

8	Hospital Risk Management	2

9	Blood Suppliers (CBS, Hema-Quebec)	9

11	MoH (Provincial)	2

13	Recipients of blood products	2

**11**	**Total**	**45**

Note: Numbers in the text after job title refer to individuals in that category interviewed.

We also planned to conduct interviews with individuals from the Ontario Hospital Association and from Health Canada (the regulator of the Canadian blood system). It became clear while talking to individuals from the Ontario Hospital Association that the association does not currently play a role in blood product recalls/withdrawals within the province. Health Canada, despite being asked on several occasions, declined to participate in the project, having been advised by their legal department not to do so in order to avoid the possibility of liability issues.

In order to obtain as comprehensive an understanding as possible of the recall/withdrawal process across the province of Ontario, we made sure that the different types of hospitals were represented (large urban teaching hospitals, smaller urban hospitals and rural hospitals). We also interviewed individuals from Héma-Québec, to get a sense of how the process compares between blood suppliers and key informants from other provinces (Alberta, British Columbia, Nova Scotia, and Saskatchewan).

The protocol was approved by the Research Ethics Board of McMaster University. The interview guide included questions on understanding the terminology involved in recall/withdrawal situations; individuals' experiences of these situations and the actions taken by different individuals and or stakeholders at different stages in the process; existing policies and procedures; questions related to disclosure of information and notification of recipients, including who should be involved in this process and how it should be done. Respondents were also asked for any suggestions they might have on improving the process (see Additional File 1: Appendix 1).

Interviews were conducted over a four month period from May to August 2006, either in person or by telephone. The interviews were conducted by two of the research team members (EA and BMcC). To maximise consistency of questioning, a semi-structured interview guide was followed in each of the interviews. The questions were open-ended and interviews lasted between 45 minutes to 1 hour. All interviews were audio-taped and then fully transcribed. The transcriptions were checked for accuracy against the tape-recordings and then imported into NVivo 7, a qualitative data management software program commonly used in qualitative research [41].

A team analysis approach was employed whereby as transcripts became available, they were read independently by several members of the research team (EA, BMcC, and JE). The team then met at regular intervals to discuss the content of interviews and to identify the themes and issues emerging from the data. A schematic for coding the data (identifying discrete passages of text that contain the same idea) was developed by EA and BMcC and then this coding scheme was applied to a new batch of interviews by EA, BMcC and JE. Inter-rater agreement was then calculated and was found to be very high (100% for major codes and 94.3% for minor codes) thus indicating that the coding scheme was working well. This schematic was then used to code the entire data set by BMcC, so that all interviews were coded using the same coding themes. This schematic acted as a taxonomy, classifying and organizing the complexity of the 45 responses and there is a close parallel between the taxonomy (reported in [38]) and the interview guide. Further, deeper coding of the data generated other propositions, which may be seen as second order constructs (see [42-44]). The analytical development of these constructs allowed relationships to be identified between the codes in the taxonomy (see [45]). These also emerged because of our interest in blood risk management. Thus the issues of notification, response to minimal risk, responsibility and legal requirement are transformed into those appearing in the results section, guided by themes and theories of risk management and expert judgement outlined above, i.e. the heuristics and logics used to deal with uncertainty and to manage risk for protective, liability and precautionary reasons. In presenting the findings we have selected quotes that illustrate these ideas, allowing as many respondents as possible to speak. We suggest, therefore, that our paper makes a methodological contribution by using the typifications of social phenomenology to transform respondents' concerns into specific risk discourses.

## Results

### Uncertainty as risk: What do we call what just happened?

As a foreshadowing to outline different conceptions of how blood product risk should be managed, we note confusion among respondents over the meaning of the terms 'recall' and 'withdrawal'. There is meant to be clarity in:

#### Recall

"With respect to a health product, other than a medical device, means a responsible party's removal from further distribution or use, or correction, of a distributed product that presents a risk to the health of customers or violates legislation administered by Health Products and Food Branch Inspectorate (HPFBI)" [46].

#### Withdrawal

"The removal from further distribution or use, or correction of a distributed product where there is no health and safety risk and no contravention of the legislation administered by the HPFBI. It is not considered to be a recall" [46].

Yet only 14/45 (30%) of individuals interviewed indicated that they knew the difference between the two terms. The difference between the terms is confusing for both hospital and blood supplier personnel:

*"I think they are confusing. The only reason, I'll be honest; the only reason that I'm familiar with it is because of the incident that we went through ..." *(Laboratory Manager-01)

*"Well ... it's very confusing. We are not exactly sure when they recall something or withdraw something. It's, you never know why they are asking you to return something. And they don't give you any extra information, and it is somewhat frustrating, because you know why are they doing this?" *(Lab Technologist-06)

*"Um... gosh... there probably is a very important difference and I ... I would be lying to you if I said with confidence that I could tell you the difference" *(Physician using blood products-01)

The confusion is mainly associated with diagnosis (what is it?) and does not appear to influence the action (what is to be done?) taken to deal with the recall or withdrawal notification at the hospital level. Regardless of whether a recall or withdrawal is issued, the initial action taken by the hospital Transfusion Service is the same: implicated products are removed from useable inventory. So would uncertainty be removed if one term was applied?

*"I don't think they should be handled differently. You know if for whatever reason a product is being taken off the market, it would, it's unfortunate that you have two terms where again they have different connotations. I think one term should suffice for all and... they should be handled in the same way." *(Physician Blood Bank-03)

*"Well I guess the fact that we have so much trouble remembering which is which... could be problematic. I think for the regulator they do require. I mean it's important to have two different terms... with two different definitions because they, they are two different matters. In practice that we get the two terms mixed up, I'm not sure it really matters*..." (Blood Supplier-07)

### Managing the risk - three conceptions of risk

While there were differences in labelling what was happening, there were none in terms of prognosis (likely outcome), seen universally as the removal of unsafe product. In other words, expert opinion converges with respect to the goal - safe blood - but on how and why this might be done there is divergence, thus revealing differences in management perspectives. We identify three approaches to managing the risk from this organisational threat: risk as hazard, risk as liability and risk and precaution.

Risk as hazard emphasises the potential adverse consequences to patient well-being. Such an approach demands immediate removal and the full disclosure of what is happening to patients. Furthermore, it suggests that those notifying patients should be as close to the patients as possible, usually the treating or transfusing physician. This risk is almost a given and most comments refer to the importance of physician notification to allay any or all fears about hazard, the physician being seen as trusted, knowledgeable and close to the patient. There is agreement on who is central in this immediate task of ensuring blood safety, the blood supplier, but different stakeholders hold the risk as hazard view for different reasons. The blood supplier itself sees a distinctive chain of responsibility and action if blood may be unsafe - from themselves to the physician to the patient.

*"... you know we are in this era of informing the patient, but I think to some extent the pendulum can go too far ... and that we need to be careful about not giving patients information that's no of value... I think we can overdo some of the informing of patients, it's almost like we're passing the buck and not kind of letting the responsibility stop somewhere along the way with a physician..." *(Blood Supplier-07)

This discussion on the need to notify seems supported by all stakeholders. Hospitals see that any notification from the blood supplier highlights a concern and the need for action.

*"Now, I would anticipate that if the blood... supplier... was concerned enough to notify us as an organization to recall a product, then the degree of risk is always such that it would be important for us to notify the patient. You know what I mean. Like, the risk assessment has already taken place at the blood supplier" *(Hospital-Risk Management-02)

*"I guess from my perspective there is either risk or there is no risk, and if there's no risk they're not notifying us. If they're notifying us it's because there is risk*" (Hospital-PR-01)

Physicians tend to agree but see themselves and are seen as those best positioned to make patients aware of potential problems as 'they know' their patients best.

*"In my opinion, it's the role of the clinician who's caring for the patient to manage those things" *(Physician using blood products-05)

*"It's useful to have a well-informed recommendation from the Blood Supplier but the hospital always has to use its discretion and the difference there is that we know our patient population" *(Physician using blood products-07)

*"I think ultimately the physician always has that, you know, discretion. And that's clear in case law" *(Blood Supplier-04).

Others feel that the physician may not have all the skills necessary to manage risk as hazard, especially if their style is based on a confident, medical approach to the issue.

*"So I would be perfectly supportive, of a hospital system or provincial system or even a nation-wide system that developed advisory guidelines. But I don't think it's a situation where if it is ... that a physician doesn't want to notify her patients that, that somehow some other agency needs to get involved and compel that notification" *(Physician- Blood Bank-03)

*"I don't know whether there would be a place for a third party to come in, where a third party would contact the recipient and say 'I know that your physician spoke with you, or that you received a letter, this is just a reminder that you might need to go for further testing'" *(Laboratory Technician-04)

*"Well I don't know if everybody has them but a patient representative would be one option. I think somebody with those sets of skills. Like not just the technical skills but the people with counselling skills and discussion. I mean they'd have to have some kind of knowledge about blood and risk*" (Provincial Health Ministry-02)

Risk as hazard was the best articulated of the discourses on how to manage. Yet risk as liability, where the potential consequences for system integrity of specific practices were central, received greater expression from respondents. Patient well-being is of course still vital but now disclosure to patients of events that might affect their well-being protects the blood supply institution as well. Such management is seen particularly at the supplier and hospital levels. The identification of the risk and its disclosure were closely related. But under many comments lies an often implicitly stated concern over liability, i.e. what is our liability if we do not act (disclose) and something happens? In other words, their responses are anchored around current operational characteristics of the healthcare system and their legally demarcated roles within that system.

Liability is often framed in terms of outside perceptions, specifically and not unusually, on how public perceptions of expertise and expert response may be framed. In this discourse, the role of the blood supplier is central but responses to their notices may vary depending on how others see the issue and their liability. The blood supplier must always give an opinion.

*"We [Blood Supplier] would always give our opinion as to follow up." *(Blood Supplier-01)

The importance of local circumstances and their possible impact on liability are recognised by the blood supplier. It must balance its obligations with that of the independence (and discretionary action) of institutions such as hospitals and doctors.

*"What we do in our centre is... we take a look at the reason for the recall, we may provide that information. The hospital doesn't do much with it, and they know they'll get a supplementary letter from me if I think recipient notification is required so that decision whether recipient notification is required is made here. I think that ... works better in our environment because I have more experience in this than the regional lab tech ... and we don't have experienced blood bank directors in most of the blood banks." *(Blood Supplier-02)

Yet circumstances at the local level may affect response to this opinion.

*"But the Blood Supplier has their own ideas about what, in what situations do recipients need to be notified so they advise us. In our opinion, you do or do not have to notify the recipient if this product has been transfused already and we sometimes do what the Blood Supplier asks, although we're a little more aggressive about notifying patients than what the Blood Supplier does." *(Physician using blood products-01).

*"I think you know, some hospitals are in a position where they absolutely must rely on the expertise of the Blood Supplier. I mean, they, you know, primary care hospitals that I assume are somewhat more comfortable just saying, 'Look, just tell us what you want to do. Tell us what you want to say and we're not gonna do any independent analysis. Just, you know, provide us with what your recommendation is.' Other hospitals are saying, 'okay Blood Supplier, thanks for the information. We're gonna consider this independently. We have the expertise to do so, you know. The only thing we want you to do is provide as much information as you can on the risk and we're all consider it at our Transfusion Committee and we'll all decide ultimately what we think our physicians should do in terms of patient notification.' ... that kind of thing. You know, so there's quite a difference. So that is a part that we're struggling with a little bit in terms of how do we fulfill our obligations? We never want to be, we never want to fetter anyone's discretion in terms of notification. That's for sure. Though part of us is saying, you know, part of the time we think, okay, we can provide all of the, all of the information, all of the risk information as clearly as we can, and that's it and then the hospital can sort of make their decisions. But there's another component then because we don't want to be in a position of... we don't want to abdicate our responsibility and thrust the decision making on hospitals, you know, so there's sort of a tension there between those two" *(Blood Supplier-04).

The hospital is perhaps in a position of having to respond to local pressures before the full facts are known because they are local institutions and have explicit liability concerns.

*"I believe in full disclosure even if all you can say is here's what we think happened, here's why it happened, here's what we know and don't know, and here's a mechanism for either monitoring or what have you down the road. So in the absence of that, in the event that there's some new novel research finding in the not so distant future, I've never been told about this, I didn't know I had exposure, some marvellous new technology or technique comes along that would perhaps allow me to be more definitive. Well I don't even know about it to pursue that or include it in my own medical history" *(Hospital Adminstrator-02)

While some groups which are closest to the patient that is being transfused with blood see most recalls as leading to minimal risk, perhaps displaying an overconfidence in the role of scientific assessment, others are concerned about outside perceptions and what these might do to the situation.

*" ... we couldn't identify what the risks were and the risks were minimal, therefore we should not disclose, and [the Chief of Governance] was adamant that we needed to ... I was really surprised that the recommendation by the Transfusion Committee was totally disregarded" *(Transfusion Safety Officer-01)

Waiting for an assessment can lead to much time thinking through the issue. This may result in an affect response, especially with respect to the media. This may be exacerbated by public attention being roused before a system-based announcement can be made.

*"And sometimes we've got no, we don't have an assessment yet... so we're sitting ... waiting, you know and there's a time delay there so... my biggest fear is in the meantime it hits the media, now we've got an issue" *(Laboratory Manager-03)

*"But the problem is, is that you have media that's watching, and so then, then there's a different spin put on this when it hits the newspaper. You know... another bloody scandal. So are you more concerned about your public relations or are you more concerned about the effect you're going to have on patient care?" (*Laboratory Manager-01)

Much of the liability discourse takes into account the perceptions and roles of other stakeholders, both inside and outside the blood supply system. The third discourse - risk as precaution - does that too but also seems to treat problems on a case-by-case basis as uncertainty cannot be fully removed or explained. It is a view of risk often held and articulated by system administrators. A precautionary approach has been based on a changed perspective toward patients and institutional/professional partners. And precaution - taking care - is necessary as interests and ways to achieve them may be dissimilar. The blood supplier sees precaution as necessary because of these dissimilarities.

*"I don't know how much a hospital's disclosure policy would vary from one hospital to another. I mean one would hope that there's some uniformity in that or else disclosure of things is going to be a problem for very much more than recalls and withdrawals because I think there's all kind of things in a hospital that one might decided you disclose or you don't disclose" *(Blood Supplier-07)

*"I'm not looking at it in the capacity of disclosure and our disclosure policy and for us it's not as simple as just telling people something went wrong. We have to ... weigh out what the risk of telling someone versus the benefit of telling someone ... we very much are very strong proponents of disclosure and do it unless the risk of advising is significantly worse than not we certainly learn towards advising patients" *(Hospital Administrator-03)

Furthermore, the blood supplier argues for precaution because of the need to respect the different needs and sensibilities of different types of patient.

*"Because right now, the decision whether to inform a patient is based upon the doctors and the doctors in hospitals alone. The patient has no input whatsoever.... now the CJD thing was a perfect example of some people probably didn't want to know all the recalls, because what good could this have done. But some people did. So that there has to be a choice made by the patients... and my view and most patients' view on that is that they have no business making that decision for patients. Now, it's just the whole attitude of the health system, it's not anyone's particular fault, but it's just the attitude of you know, it's your responsibility and we'll make the decision. I think patients feel you know a little ticked with that" *(Transfusion Recipient-02)

Often, agencies lack information about these patients: the recipients of the transfused products.

*"We only have part of the story when we do these recalls and withdrawals which is the information from our end about the donor or about the component but we don't know anything about the recipient at the other end. And the importance of the information and about what should be done has to be determined in the context of the recipient" *(Blood Supplier-03)

*"I know our medical staff has a problem with the word recommendation because, I think it centres around the fact that they are dealing with facts about the unit and have no facts about the patient. Certainly, if you go to the level of individual patients, you can, I think justify different actions because of the different situations of patients" *(Blood Supplier-01)

Precaution on the part of local system administrators is weighed not only with respect to hazard but also institutional and patient autonomy.

*"For us it's not as simple as just telling people that something went wrong. We have to, we like to, weigh out what the risk of telling someone versus the benefit of telling someone ... so I think there needs to be a way to bring hospitals together and recognising the hospitals are independent and are free to make their own choices to the extent that that can be coordinated goes a long way because ... we have to have a plan B that while we didn't think disclosure was appropriate, in the event that another hospital chose to disclose, we had to be prepared for how we would respond to that" *(Hospital Adminstrator-03)

*"It's a very paternalistic approach to say 'oh well we know it's bad for you and so you know we want to spare you the pain of having to you now think about things like this so just let us do it.' That has not worked well in the past and we are a sort of newer generation of people where the attitude has changed... So, give the sort of transition in the social more that is out there.... I think trying to take that paternalistic approach that we will hide things from you for your own best interest it just won't sell" *(Transfusion Recipient-01)

## Discussion

In managing the possible consequences of this error, a quite minor, almost theoretical, risk to the safety of the blood supply, there was uncertainty about what was being managed - a recall or withdrawal. This has now been resolved in part through the authors' report (see [38]). The term recall is now used. Clarity in identifying responses to a possible hazard is necessary. But the then uncertainty around terminology did not stop the problem from being tackled but it caused pause for thought. We note too in this intensely regulated, safety-first environment that conventional risk discourse - as hazard - and management as its removal or mitigation did not loom large. It is a given in this tightly knit and collegial community that patient safety is paramount. There appears to be significant levels of trust between the different groups of professionals, something not always found in hazard management settings (see [47]). This trust and respect provide an excellent basis for existing and enhanced communication about blood hazards in these groups. Communication between parties with respect to the issue, what it means, and what responses are possible is key. It remains important to remember that there may be differences in approach to, say, discourse, or treatment and some adjustments may be required. And while the discourses are ones of engagement in this case, that does not necessarily mean that there will not be adversarial interactions over preferred management strategies and rationales, especially in the importance given to patient notification. (See [48] for a discussion of expertise and collaboration). This need for disclosure may in fact lead to a further risk management challenge. In our case, there was discussion among risk managers about risk amplification through disclosure. Research [49] has certainly shown the importance of the physician communicating risk issues to the public.

Risk as liability views system integrity and maintenance as an important goal. Management entails not only the use of knowledge about patient safety and system practice but also a cognitive and emotional commitment to the aims and goals of the organisation. The supplier agency has created that commitment and loyalty within a changing Canadian health care system (see [37]). Yet uncertainty remains and this may be due to the centrality of precautionary principles in the blood supply system. Thus, it is not surprising that risk and precaution are seen simultaneously as two dimensions of hazard management. Good record keeping and monitoring of transfused blood (and its recipients) enables precaution to be central in risk management. Kaplan et al [50] advocate a medical event reporting system for transfusion and we concur with their suggestions. Such a system (Transfusion Error Surveillance System - TESS) is currently being developed and piloted in Canada [9]. Furthermore, the difference discourses in expert groups within the same decision-making system (but likely at different levels of decision-making) may be beneficial, if recognised as such. 'Hazard' is a safety-first approach protective of health; 'liability' ensures that the challenges of risk amplification and perceived injury may be dealt with if an unknown risk is disclosed and 'precaution' ensures due diligence and quality assurance before action is taken.

With respect to the three risk discourses, some are more likely to be articulated by some professional groups - hazard by physicians, liability by administrators (hospital and blood supplier), precaution by administrators (especially the blood supplier). Those groups are largely responsible for managing the risk from these respective vantage points. There is largely a coherence of expertise within those realms, although it is in part challenged by laboratory technicians (hazard and liability) and transfusion recipients (liability and precaution). A vital strategy in managing any risk or consequences of error is a coherent response. This coherence may break down as different dimensions of risk management are required (patient safety, system maintenance) or different professional and lay groups engaged. In fact, it is possible for different discourses to be used by one or more different groups. In all discourses, it is possible for different heuristics to guide management response, overconfidence with respect to medical expertise and the scientific assessment of risk (physicians and blood supplier), anchoring to the legal and formal positions of institutions (hospitals), and affect with respect to feelings about uncoordinated public announcement of a risk and perceived media response (laboratory technicians and transfusion staff). The blood supplier uses all discourses because of its variable role in supplying safe products.

## Conclusions

In adopting a quasi-foundational approach, there are ways to ensure rigour and the trustworthiness of interpretations. The social phenomenology of Schutz, used to derive themes and as a basis for theory development from respondent perceptions, requires meeting three postulates, namely logical consistency, subjective interpretation and adequacy. For the first, we have highlighted how the research problem and methods were derived from a real world problem on which was based the questionnaire, sampling strategy and the need to interview; for the second, by using respondents' views to develop interpretation (different than most studies as the first order or naturalistic constructs are expert ones); and for the third, by linking second order constructs to activities and phenomena in blood risk management. Furthermore, trustworthiness can be demonstrated by credibility and confirmability (see [51,52] for practice-based examples). We have provided a trail from problem identification, questionnaire development, coding taxonomies to themes and theoretical development. We have also provided the rationale of respondent selection and sampling design as well as an outline of procedures followed to arrive at constructs or themes. We have derived through these themes risk discourses and logics relevant to blood risk management. Before finalization, the study results and recommendations were presented at a consensus conference of study participants and stakeholders, to ensure validity of data interpretation. All suggestions made were incorporated into the final results. Although we recognize that all researchers will not necessarily agree to the ways we have sought validity for these findings (see [53]), first author conversations with risk managers point to the utility of the interpretations. Thus this qualitative investigation has contributed a nuanced risk characterization.

In fact, all discourses are necessary to manage this low-level risk. This coherence and differentiation of expertise around managing blood risk has practical consequences. As Hunt [54] notes, governance of risk is characterised by risk avoidance rather than risk management and is in turn dominated by a preoccupation with safety. The further corollary is the expanding panoply of regulation and guidelines. This may be noted in blood supply. And when 'something happens'--a threat to safety--the virtuous cycle of risk, regulation, and prescription is interrupted. The relentless pressure for the systemisation and integration of risk management practices continues as a watchword for corporate social responsibility (see [55]). This virtuous cycle is reinforced by the use of a precautionary logic. As Haggerty [14], notes, precaution emphasises the worst eventualities. It is not so much about risk. It "invites one to anticipate what one does not yet know, to take into account data, hypothesis and simple suspicions" [56:288]. With a product as vital and special as transfused blood, precaution is necessary. But societally it may feed anxieties and increase risk aversion. Practically, we point to consideration being given to enterprise risk management (ERM), recently developed as ISO 31000 (see [57]) and accepted by the Canadian Standards Association in 2010.. ERM considers any risks or uncertainties affecting objectives, requires a flexible organization to tailor risk management, formalising monitoring review and consultation, and demands accountability from all those dealing with the risk. All senior managers must be committed to the process which must be used by all decision-makers in a flexible organizational structure. In its stages, it considers risk in careful ways. In setting the context and identifying risk, it suggests consideration of risk appetite and triggers. In analyzing and treating risk, ERM points towards acceptance, control and mitigation. It suggests ensuring that residual risk and its potential impacts are not ignored as it is not possible to remove all uncertainty. ISO 31000 may be complemented by using a safety-case approach which requires the incorporation of all evidence to ensure the system is safe to operate (see [58], potentially modified to permit inputs from all appropriate stakeholders to organize heterogeneous information and concerns to ensure the safety and dependability of a larger network (i.e. the blood system).

Elements of the blood management system have been drawn to ISO 31000. Furthermore, processes such as recording, inter-professional communication, notification and disclosure have been applied in dealing with blood risk. Risk triggers and residual risk point to the relevance of hazard and liability discourses, especially as in other domains, use of the precautionary principle has been shown to trigger concerns and lower trust in governance structures (see [59]). So for the institution of precaution we must be clear on what we are managing, why and in what ways. For the Canadian blood system, a new procedure of providing reasons for recall will assist hospitals and other donation agents in managing perceived risks. Yet the role of expert biases and domain interests are likely to continue to exist, and must be understood and incorporated to ensure blood safety and continued public trust and to provide timely responsiveness in such a high reliability system. We suggest the framework of ISO 31000 emphasising context, risk identification and assessment, risk treatment, communications and consultation is useful, along with a safety-case approach. Communication about ways to protect public safety is always necessary and this must include clarity on definitions, responsibilities, and public perceptions and what the consequences of even minor errors mean in a complex system. Furthermore, error as a risk state needs careful theorizing and application in systematic risk management approaches.

## Competing interests

The authors declare that they have no competing interests.

## Authors' contributions

All authors contributed to data collection and analysis. JE carried out the main interpretations of the data. JE and NH wrote most drafts of the paper. EA commented on drafts of the paper. All authors approved the final manuscript.

## Pre-publication history

The pre-publication history for this paper can be accessed here:

http://www.biomedcentral.com/1471-2458/11/666/prepub

## Supplementary Material

Additional file 1 **Appendix 1: Interview guide for understanding the management of blood products in Ontario**. Appendix provides the questionnaire used to explore risk management of blood products under conditions of uncertainty. Click here for file
