# Supplementary material for: Do expert assessments converge? An exploratory case study of evaluating and managing a blood supply risk
Source: BMC Public Health. 2011 Aug 24;11:666. doi: 10.1186/1471-2458-11-666 (PMC3223866; doi:10.1186/1471-2458-11-666)
Supplement: Additional file 1 — Appendix 1: Interview guide for understanding the management of blood products in Ontario. Appendix provides the questionnaire used to explore risk management of blood products under conditions of uncertainty. [file 1471-2458-11-666-S1.DOC]

**Appendix 1: Interview Guide**

**Improving the Process of Recalls/Withdrawals of Blood Products in the Province of Ontario: A POLICY STUDY**

Thank you for accepting our invitation to talk with us today.

To review, in this study we are gathering information about occasions when blood products have to be recalled or withdrawn. We would like to ask you some questions about these issues with the purpose of seeing if the present process works well or if it should be modified in some way.

Do you have any questions before we proceed?

I have a few reminders before we start the interview:

- Please answer in general terms and if you use examples please don’t name individuals, institutions or places.
- Remember that you can refuse to answer any of the questions in the interview or withdraw from the interview at any time.

Thank you for your consent forms/demographic questionnaire . . .

I see from your demographic questionnaire that you are. . .

What is your understanding of the **terms recalls and withdrawals** related to blood products?

- Are these terms appropriate?
- Do you think there is any **confusion** between the terms?

[Note to interviewers:

- Recall= the removal from further distribution or use of a product that violates legislation administered by Health Canada
- Withdrawal = the voluntary removal by the manufacturer from further distribution or use of a product that does not violate legislation administrated by Health Canada].

Have you had **direct experience** with a blood recall or withdrawal?

- if yes, did it pose a **theoretical or known** risk to recipients?
- Do you want to elaborate on the experience?

**Hospital questions**

Does your hospital receive notifications from the blood supplier about **all** recalls/withdrawals or just notifications that are **relevant** to product lot numbers that your hospital has received?

- If you receive **all** notifications, **how and who** prioritizes or determines which notifications are relevant for your hospital?

Can you describe what happens (i.e. the process that your institution follows) when your institution is informed that a product has to be recalled / withdrawn?

PROBES: what happens to the products that are being removed and does or should CBS give instructions on what should be done?

- **Destroyed** Immediately?
- **Quarantined?**
  - If Quarantined, **when and who** makes the decision to either **use** the product or **destroy** the product?
  - How is that decision made? (i.e. based on product expiry dates, further instructions from CBS)

Do you handle recalls and withdrawals in **different** ways? OR Based on your understanding of the difference between recalls and withdrawals, do you think they **should** be handled differently?

How many recipients do you deal with in the average month? Do you think that the recall/withdrawal **processes vary depending on the number and type** **of recipients** potentially affected? Should they vary?

**IF THIS IS NOT MENTIONED ABOVE, ASK:**

Does your institution have a **policy and procedure** (an SOP?) for handling recalls or withdrawals?

- For you, what are the **key elements** of this policy / procedure?
  - PROBE: clarity, safety, emphasis, flexibility.
- Do you have any **documents** relating to these that we might be able to see?
  - If so, at the end perhaps we can come back to this to discuss how we would obtain them?

How **frequently** have these policies / procedures been used? OR when was the **last time** that these were used?

**Blood supplier questions**

In your opinion, if a recall or withdrawal is necessary, **to whom** should the Blood Supplier disclose information about the recall or withdrawal?

- **What information** should be disclosed?
- Should **ALL information** be disclosed?
- If not, why not?
- If not all, **what information** should they disclose?
- Should **recalls/withdrawals be handled differently**?

Do you think that the **Blood Supplier** has an obligation to disclose **ALL of the information** regarding a recall or withdrawal to **hospitals**?

- If not, why not?
- If not all, **what information** should they disclose?

How important do you think it is for the **Blood Supplier** **to explain the risk** associated with the recall/withdrawal?

- What if the risk is **not yet known**?

How important is **‘trust’** between the **blood supplier and hospitals** for the recall/ withdrawal process to be effective?

- Does the current level of trust need **to be improved**?
- If so, how can trust be **improved**?

When the **Blood Supplier** notifies hospitals of a recall or withdrawal, should the information be **accompanied by a recommendation** whether or not to notify recipients about the recall?

At the **hospital level, who do you think should be involved** in the recall or withdrawal?

Probe: The Transfusion Committee; The Medical Director of Transfusion Medicine; The

Medical Advisory Committee; The Hospital CEO, The hospital risk management team, patient safety departments, legal department, ethicists, regulatory departments

In some hospitals, the transfusion specialists also work for the blood supplier. Do you see a potential conflict of interest in the decision making process around recalls / withdrawals in this case?

**Notification questions**

In your opinion, who should make the **decision to notify patients** if they have been transfused with blood products that are subsequently recalled or withdrawn?

- Should this decision be the **same for all hospitals** within a province or should each hospital make their own decision, which could potentially result in a situation where hospitals may have different approaches?
- If the decision regarding notification is not made by individual hospitals, do you have any thoughts on how **potential conflicts** between this decision and the individual hospital’s disclosure policy should or could be managed?
- If individual hospitals are allowed to make their own decisions, do you think there is a potential problem with different hospitals making different decisions?

In your hospital, who **currently** makes the decision whether or not patient notification is necessary?

- Who do you think **should** be involved in this decision making process?

Probe: The Transfusion Committee; The Medical Director of Transfusion Medicine; The

Medical Advisory Committee; The Hospital CEO, The hospital risk management team, patient safety departments, legal department, ethicists, regulatory departments

Probe if appropriate: are you aware that blood products can be recalled or withdrawn by CBS due to errors in manufacturing which can cause no harm whatsoever to recipients? (e.g wrong pipettes used during manufacturing process) Would or should such a scenario of minimal risk have an impact on the decision to notify patients?

In your hospital, once a decision has been made to notify recipients about a recall/withdrawal, does the **hospital** **notify patients directly** or does the hospital notify **patient’s attending/family physicians**?

- In your opinion, who should be notifying patients: hospital or physician?

**Other agencies questions**

It is likely that decisions about recalls/withdrawals may involve **various agencies**.

- Which **other agencies** should be involved?
- How should their **interaction** be managed?
- Which one should be the **lead agency?**
- Which **risk management protocol** should be followed?
- **What if these procedures conflict**?

ASK IF NOT ALREADY MENTIONED:

What role if any should the **Provincial Public Health Department / Ministries of Health** / Health Canada / provincial blood coordinating office?

In your opinion, should the **media** be involved in recalls/withdrawals?

If yes, in what way?

- At what point should the media be informed?
- Should the media be informed about **every** recall/withdrawal?
- **Who** should inform the media?
  - - CBS, hospital public relations?
- Should the media **only** be informed of a recall/withdrawal if **patient notification** is going to occur?
  - - What information should be given to the press?
    - Should the media only be informed **after patients have been notified**?

If no, why not?

Do you think that the fear of **negative media publicity** has any impact on the decision making process around the handling of recalls or withdrawals?

In your opinion, once a decision has been made with regard to the specific handling of a recall or withdrawal issue **could** **negative media pressure alter that decision**?

- **Should negative media pressure alter that decision?**

Do you have any general **suggestions for improving the recall/withdrawal process**:

- for the Blood Supplier
- for hospitals
- for other agencies we have talked about? (e.g Public Health?)

Probe: How important are the following in developing a recall / withdrawal process? A) Numbers of those affected? B) Uncertainty of risk? C) Maintaining public confidence in supply and agencies?

ASK IF APPROPRIATE:

A **general question** to end with - What kind of risks do you see blood transfusions posing to Canadians today? Do you see those as major risks?

Is there **anything else** that you would like to say that we have not already covered?

- Is there anything that you **expected** us to ask that we did not ask?

If necessary ask:

- that copies of any available documents be sent to MTRP
- For any additional names of individuals to approach for interview
